# Comparison of early outcomes between unicompartmental and total knee arthroplasty in patients with anteromedial compartment knee osteoarthritis and class II obesity: a retrospective case analysis

**DOI:** 10.3389/fsurg.2025.1616382

**Published:** 2025-08-04

**Authors:** Qingwei Li, Shenhao Zhai, Zongyou Mu, Xubin Zhang

**Affiliations:** 1 School of Clinical Medicine, Shandong Second Medical University, Weifang, China; 2 Department of Orthopedic, Qilu Hospital Dezhou Hospital of Shandong University Dezhou Hospital, Dezhou, Shandong, China

**Keywords:** anteromedial osteoarthritis (AMOA), class II obesity, unicompartmental knee arthroplasty (UKA), total knee arthroplasty (TKA), early functional outcomes

## Abstract

**Background:**

This study compared early clinical outcomes of unicompartmental knee arthroplasty (UKA) and total knee arthroplasty (TKA) in patients with anteromedial osteoarthritis (AMOA) and class II obesity during postoperative hospitalization and at 1, 6, and 8 months postoperatively.

**Methods:**

A retrospective analysis was conducted on 85 patients with class II obesity who underwent UKA (*n* = 45) or TKA (*n* = 40) between January 2022 and January 2024. Perioperative parameters, including operative time, incision length, hemoglobin and albumin levels on postoperative day 2, and hospital stay, were compared. Functional outcomes were assessed using the visual analog scale (VAS), Hospital for Special Surgery (HSS) knee score, and range of motion (ROM) before surgery and at 1, 6, and 8 months postoperatively. Complication rates were also recorded.

**Results:**

All patients completed surgery successfully. The UKA group had significantly shorter operative times, smaller incisions, higher postoperative hemoglobin and albumin levels, and shorter hospital stays (*P* < 0.01). At 1 and 6 months, UKA patients showed better VAS, HSS scores, and ROM (*P* < 0.05). By 8 months, no significant differences remained. Complication rates were lower in the UKA group (2.22%) than in the TKA group (17.5%) (*P* < 0.05).

**Conclusion:**

Both UKA and TKA improve pain and function in AMOA patients with class II obesity. However, UKA offers advantages in perioperative recovery, early functional outcomes, and complication rates, supporting its use in appropriately selected patients.

## 1 Introduction

Anteromedial osteoarthritis (AMOA) is a common degenerative knee disease characterized by osteophyte formation, reduced cartilage repair, and chronic weight-bearing wear. Consequently, these changes progressively thin and degrade articular cartilage, narrow joint space, and ultimately damage the cartilage surface in the anteromedial compartment of the knee (1).

Since the 1980s, the global prevalence of obesity has increased dramatically, nearly doubling worldwide (2). According to the World Health Organization, body weight is classified based on the body mass index (BMI), with BMI > 25 kg/m² defined as overweight and BMI > 30 kg/m² defined as obesity. Obesity is further categorized into three grades: class I obesity (BMI 30–34.99 kg/m²), class II obesity (BMI 35–39.99 kg/m²), and class III obesity or morbid obesity (BMI ≥ 40 kg/m²). Recently, a new category termed “super obesity” has been introduced, referring to individuals with a BMI > 50 kg/m^2^ (3, 4).

Obesity is a major risk factor for knee osteoarthritis (KOA): each 5-unit BMI increase raises KOA risk by approximately 35%, more pronounced in females (5).

In early anteromedial osteoarthritis (AMOA; Outerbridge grades I–II: cartilage softening and swelling or partial-thickness fissures ≤0.5 inch not reaching subchondral bone), non-pharmacologic interventions, weight management, structured periarticular muscle strengthening, and lifestyle modification are preferred (6). Conversely, in end-stage knee osteoarthritis (KOA; Outerbridge grades III–IV: fissures >0.5 inch reaching subchondral bone or full-thickness cartilage loss exposing subchondral bone), unicompartmental (UKA) or total knee arthroplasty (TKA) is the mainstay, relieving pain and restoring joint function with prosthetic components, thereby significantly improving quality of life (7).

Previous studies indicate that medial compartment involvement predominates in patients with obesity and KOA due to greater biomechanical knee loading (8). Although TKA provides complete reconstruction, it requires resection of all articular cartilage and cruciate ligaments, compromising normal structures and potentially reducing joint stability. Moreover, patients with obesity face higher risks of intraoperative trauma and postoperative complications (9).

Advances in minimally invasive techniques and endoprosthesis design have broadened UKA indications and increased its use in obese patients. However, its clinical efficacy in moderate obesity with AMOA remains controversial. This study compares postoperative functional recovery, complication rates, and prosthesis stability between UKA and TKA in patients with AMOA and class II obesity during postoperative hospitalization and at 1, 6, and 8 months postoperatively, providing evidence to guide surgical choice. We retrospectively analyzed clinical data of patients with class II obesity and KOA treated at Qilu Hospital of Shandong University (Dezhou Branch) between January 2022 and January 2024, comparing early UKA and TKA outcomes. The results are presented below.

## 2 Methods

### 2.1 Inclusion and exclusion criteria

#### 2.1.1 Inclusion criteria

Inclusion criteria included medial compartment osteoarthritis diagnosed as Kellgren–Lawrence grade ≥3 on imaging and confirmed by clinical evaluation, with intraoperative assessment of patellar cartilage wear per the Outerbridge classification; Only patients with Outerbridge grades III–IV (cartilage fissures >1.5 cm extending to subchondral bone with bone exposure and erosion) were included; those with grades 0–II were exclude; knee flexion contracture ≤15°, varus deformity ≤15°, and range of motion ≥90°; intact function of major intra-articular ligaments, including the anterior cruciate ligament, posterior cruciate ligament, and collateral ligaments; failure of conservative treatment (e.g., pharmacologic therapy, physical therapy, functional exercise) for at least 3 months; Body mass index (BMI) between 35.0 and 39.9 kg/m², consistent with class II obesity.

#### 2.1.2 Exclusion criteria

Exclusion criteria included the presence of other inflammatory joint diseases such as rheumatoid arthritis or gout; active site of infection; severe systemic comorbidities that impair surgical tolerance, including hypoproteinemia, cardiac insufficiency, or advanced hepatic/renal dysfunction; history of previous knee surgery; and incomplete follow-up data or loss to follow-up.

### 2.2 Collection of patient data

This retrospective study was conducted based on the review of medical records from the hospital information system (HIS) and imaging archives of Qilu Hospital Dezhou Branch of Shandong University. Data were independently extracted by two trained reviewers using a predefined case report form; any discrepancies were resolved by consensus under the supervision of a senior investigator following a calibration session to ensure uniform interpretation of study variables.

The below data were extracted.

#### 2.2.1 Demographic and baseline information

Age, sex, body mass index (BMI), affected side (left or right), and comorbidities such as diabetes and hypertension. Inclusion was limited to patients diagnosed with AMOA and classified as having Class II obesity (BMI 35.0–39.9 kg/m²). Moreover, patients were assessed for preoperative joint conditions, including knee flexion range, varus deformity, and ligament integrity.

#### 2.2.2 Perioperative clinical data

Surgical method [unicompartmental knee arthroplasty (UKA) or total knee arthroplasty (TKA)], operation time (minutes), intraoperative blood loss (ml), incision length (cm), type of anesthesia, length of hospital stay (days), and perioperative laboratory results including hemoglobin and albumin levels on postoperative day 2. Intra- and post-operative complications (e.g., deep vein thrombosis, infection, wound healing issues) were also recorded.

#### 2.2.3 Postoperative follow-up data

Clinical outcomes were evaluated using the Visual Analog Scale (VAS) for pain, Hospital for Special Surgery (HSS) knee score, and range of motion (ROM) at preoperative baseline, during postoperative hospitalization, and at 1, 6, and 8 months postoperatively. Follow-up duration ranged from 8 to 12 months; 12-month assessments were only performed in patients who returned per medical advice after the 8-month visit. On postoperative day 2, standard anteroposterior and lateral radiographs were obtained to assess component positioning and mechanical alignment; evaluate the cement mantle integrity, joint space, and periprosthetic bone status; and facilitate early detection of prosthesis loosening, malalignment, periprosthetic fractures, or bone resorption, thereby ensuring optimal surgical outcomes and guiding subsequent rehabilitation planning. The occurrence of any reoperation or revision procedure was also documented.

### 2.3 General clinical data

This retrospective cohort study included 85 patients with AMOA and class II obesity (BMI 35.0–39.99 kg/m²) who underwent either UKA or TKA at Qilu Hospital of Shandong University, Dezhou Branch, between January 2022 and January 2024. All patients were selected based on strict inclusion and exclusion criteria. Patient demographic and clinical data were retrieved from the HIS, imaging records, and operative notes. All surgical procedures were performed by a single senior orthopedic surgeon with extensive experience in knee arthroplasty, thereby minimizing variability in surgical technique and postoperative management. Patients were retrospectively categorized into two groups based on the type of surgery received: 45 and 40 cases in the UKA and TKA groups, respectively. Group sizes were determined naturally according to the number of eligible patients who underwent each surgical procedure without intentional balancing or matching. No selective inclusion or exclusion was performed to equalize group sizes. This study adhered to the principles of the Declaration of Helsinki and received approval from the Institutional Medical Ethics Committee, and the ethics protocol and approval can be provided upon request.

### 2.4 Surgical methods

#### 2.4.1 UKA group

Under combined spinal-epidural anesthesia, patients were placed in the supine position. An inflatable tourniquet was applied to the proximal thigh and its distal end secured with adhesive tape in a circumferential fashion. The foot and ankle were disinfected with povidone-iodine solution in four sequential scrubs. An assistant wearing sterile gloves elevated the leg by lifting the foot. The surgeon then disinfected the remaining leg skin with povidone-iodine in four sequential scrubs. Sterile auxiliary materials were then applied, such that, except for the surgical field, everything remained beneath and behind this sterile drape. A final wipe of the surgical area with povidone-iodine was performed immediately before incision. Subsequently, the disinfected field was swabbed once with medical alcohol and covered with a transparent adhesive incise drape. A medial parapatellar approach was used, with a skin incision starting from the superomedial edge of the patella and extending distally approximately 3 cm beyond the joint line to the medial edge of the tibial tubercle. The skin and soft tissues were incised in layers. The infrapatellar fat pad was partially excised to fully expose the medial tibial plateau. An intraoperative examination of the anterior cruciate ligament (ACL) was performed to confirm its integrity. Furthermore, osteophytes on the medial femoral condyle and the medial and lateral edges of the intercondylar notch were removed using an osteotome or rongeur, along with larger osteophytes on the superior and inferior poles of the patella. A spacer was used to assess the medial compartment gap and select an appropriate femoral prosthesis size. The tibial cutting guide was proximally connected to the spacer via a G-clamp and distally fixed at the ankle. With a Z-shaped retractor protecting the medial collateral ligament, a vertical osteotomy of the proximal tibia was performed using a reciprocating saw, followed by a horizontal cut using a narrow saw blade. Subsequently, a femoral intramedullary guide rod was inserted through an entry point approximately 1 cm anterior to the upper medial corner of the intercondylar notch. A drill guide was used to create 4 mm and 6 mm holes at the distal femur, after which a posterior condylar cutting guide was installed to assist with femoral osteotomy. The posterior femoral condyle was resected with a narrow saw blade, and the medial meniscus was excised. The distal femur was then trimmed to remove residual marginal osteophytes and improve endoprosthesis fit. A combined osteophyte remover was used to contour the anterior femoral condyle with a burr, and the posterior condyle was cleared with a curved osteotome to ensure proper gap balancing. Additionally, a femoral trial component was inserted, and the flexion gap was reassessed to confirm joint balance. The tibial trial tray was fixed to the osteotomy surface using specialized fixation pins. A keel slot was created along the guide groove using an oscillating saw, and the groove was cleaned with a wedge-shaped osteotome to remove bone debris. Following thorough irrigation of the surgical field, bone cement was prepared, and the tibial component, femoral component, and polyethylene insert were implanted in sequence; the UKA group received the Oxford Phase III medial unicompartmental knee system (Zimmer Biomet). After complete cement curing, the surgical area was irrigated again. The joint capsule was sutured, the tourniquet was released, and subcutaneous tissues and skin were closed in layers. A pressure dressing was applied to the incision (10, 11).

#### 2.4.2 TKA group

Under combined spinal-epidural anesthesia, patients were placed in the supine position. An inflatable tourniquet was applied to the proximal thigh and its distal end secured with adhesive tape in a circumferential fashion. The foot and ankle were disinfected with povidone-iodine solution in four sequential scrubs. An assistant wearing sterile gloves elevated the leg by lifting the foot. The surgeon then disinfected the remaining leg skin with povidone-iodine in four sequential scrubs. Sterile auxiliary materials were then applied, such that, except for the surgical field, everything remained beneath and behind this sterile drape. A final wipe of the surgical area with povidone-iodine was performed immediately before incision. Subsequently, the disinfected field was swabbed once with medical alcohol and covered with a transparent adhesive incise drape. A medial parapatellar approach was used, with a midline skin incision beginning at the superior pole of the patella and extending distally to the level of the tibial tubercle. The suprapatellar bursa and joint capsule were incised, and the patella was everted or retracted laterally to fully expose the distal femur and tibial plateau. Partial resection of the infrapatellar fat pad and synovial tissue was performed. The anterior and posterior cruciate ligaments, menisci, hypertrophic synovium, and osteophytes were completely excised. Soft tissue release was conducted as necessary based on joint space assessment. Furthermore, a femoral intramedullary alignment rod was inserted according to preoperative planning. After setting the desired valgus angle, the distal femur was cut, and the amount of bone resected was measured to determine the appropriate femoral component size. Femoral condylar and intercondylar osteotomies were then performed sequentially. With the assistance of a Hoffman retractor, the tibial plateau and femoral condyles were dislocated, and tibial osteotomy was performed using an extramedullary guide. Throughout the procedure, the patellar tendon, collateral ligaments, popliteus tendon, and neurovascular structures were carefully protected. After patellar eversion, peripheral osteophytes and hypertrophic synovium on the patella were removed. Trial components were inserted to assess the flexion and extension gaps. Soft tissue release was performed incrementally until satisfactory gap balance and joint stability were achieved. Following thorough irrigation of the bone surfaces, bone cement was prepared, and the tibial and femoral components were implanted sequentially, along with a suitable thickness polyethylene insert; the TKA group received the Vanguard PS posterior-stabilized knee system (Zimmer Biomet).After cement curing, knee stability and range of motion were re-evaluated. The joint cavity was then irrigated thoroughly. The quadriceps tendon and patellar tendon expansion were sutured layer by layer, followed by closure of the subcutaneous tissue and skin. A sterile dressing was applied to the surgical site, marking the end of the procedure. Acrylic resin bone cement (Zimmer Biomet) was used for both the TKA and UKA groups.

### 2.5 Postoperative management

No postoperative drainage tubes were placed in either group. All patients received routine postoperative treatment, including anti-swelling therapy, analgesia, and prophylactic antibiotics (cefuroxime sodium 1.5 g IV every 12 h for 48 h). During hospitalization, subcutaneous injections of low-molecular-weight heparin sodium (0.3 g daily) were administered for anticoagulation. Upon discharge, patients were switched to oral rivaroxaban (one tablet per day) for 15 consecutive days. On the second postoperative day, patients were encouraged to begin weight-bearing ambulation under supervision.

### 2.6 Observational indicators

The primary outcome measures included operative time, hemoglobin and albumin levels on postoperative day 2, incision length, and length of hospital stay in both groups. In addition, the incidence of complications during hospitalization and throughout the follow-up period was recorded. Knee function was evaluated using the VAS for pain, the HSS score—which encompasses joint pain, function, range of motion, muscle strength, and flexion deformity—and the ROM, including flexion, extension, and abduction. These functional parameters were assessed and compared at baseline (preoperatively) and at 1, 6, and 8 months postoperatively to comprehensively evaluate the recovery process.

### 2.7 Statistical analysis

All data were analyzed using IBM SPSS Statistics for Windows version 25.0 (IBM Corp., Armonk, NY, USA) statistical software. Continuous variables were expressed as mean ± standard deviation (x¯ ± s), and comparisons between groups were performed using the independent-samples t-test. Categorical variables were presented as frequencies and percentages, and intergroup comparisons were conducted using the chi-square (*χ*²) test. A *p*-value of < 0.05 was considered statistically significant. An *a priori* power analysis was performed using G*Power 3.1 to detect a between-group difference of 5 points in HSS score (SD≈8) at six months with *α*=0.05% and 80% power requires 36 patients per group, allowing for 15% loss to follow-up yielded a target sample size of 85.

## 3 Results

### 3.1 Patient enrollment

**Figure 1:**
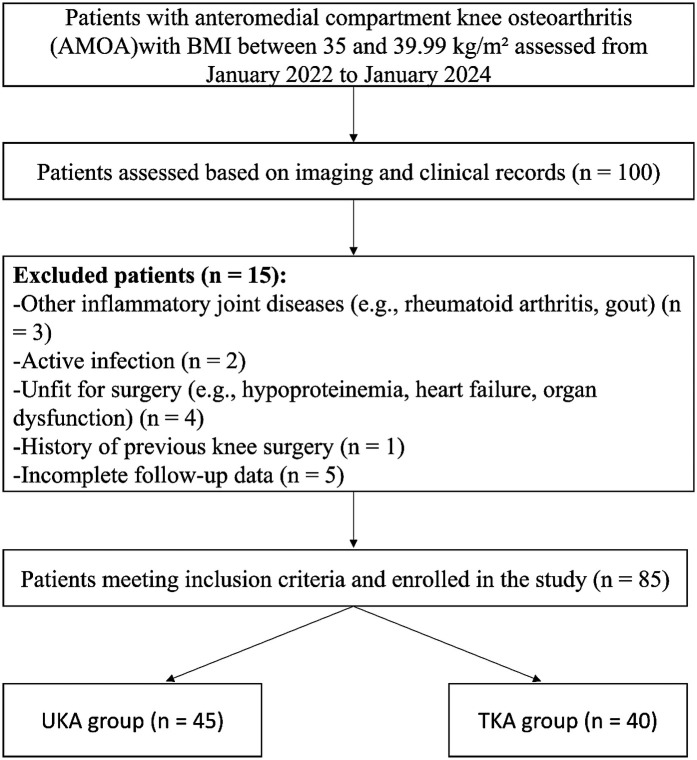
Case selection process of this retrospective study. Among 100 patients with anteromedial compartment knee osteoarthritis (AMOA) and BMI between 35 and 39.99 kg/m^2^ assessed between January 2022 and January 2024, 85 patients met the inclusion criteria and were divided into two groups based on the surgical procedure received.

All patients diagnosed with AMOA and classified as Class II obesity (BMI 35–39.99 kg/m²) who underwent either UKA or TKA between January 2022 and January 2024 were screened for eligibility. After applying the predefined inclusion and exclusion criteria, a total of 102 patients were initially identified. Of these, 17 patients were excluded due to inflammatory joint disease, previous knee surgery, incomplete clinical data, or loss to follow-up. Ultimately, 85 patients met the inclusion criteria and were included in the final analysis. The patient selection process is illustrated in Figure 1.

### 3.2 Comparison of baseline characteristics

A total of 85 obese patients were included in this study and divided into two groups according to the type of surgery received. The UKA group consisted of 45 patients (16 males, 29 females), aged 52–74 years, with a mean age of 64.28 ± 5.44 years and a mean BMI of 37.68 ± 8.22 kg/m². The TKA group included 40 patients (15 males, 25 females) aged 54–75 years, with a mean age of 64.23 ± 6.23 years and a mean BMI of 37.23 ± 1.36 kg/m². There were no statistically significant differences in baseline characteristics between the two groups (*P* > 0.05, Table 1).

**Table 1:** Preoperative baseline characteristics of patients in the Two groups.

Variable	UKA group (*n* = 45)	TKA group (*n* = 40)	Test statistic	*P*-value
Sex (male/female)	16/29	15/25	*χ*² = 0.06	0.81
Age (years)	64.3 ± 5.4	64.2 ± 6.2	t = 0.04	0.97
BMI (kg/m²)	37.4 ± 1.8	37.2 ± 1.4	t = 0.58	0.57
Affected side (left/right)	18/27	18/22	χ² = 0.29	0.56
Diabetes (yes/no)	12/33	10/30	χ² = 0.05	0.82
Hypertension (yes/no)	19/26	21/19	χ² = 0.29	0.59

BMI, body mass index; n, number; T, t-value; TKA, total knee arthroplasty; UKA, unicompartmental knee arthroplasty; χ², chi-squared.

### 3.3 Comparison of perioperative clinical data

The UKA group showed significantly better outcomes in several perioperative indicators compared to the TKA group, including less intraoperative blood loss, shorter operative time, smaller incision length, shorter hospital stay, and significantly higher hemoglobin and albumin levels on postoperative day 2 (all *P* < 0.05, Table 2). These findings suggest that UKA is associated with less surgical trauma, reduced metabolic stress, and faster postoperative recovery.

**Table 2:** Comparison of general surgical data between the Two groups.

Group	Intraoperative blood loss (ml)	Operation time (min)	Incision length (cm)	Hospital stay (d)	Hemoglobin on postoperative day 2 (g/L)	Albumin on postoperative day 2 (g/L)
UKA group	63.03 ± 4.76	66.03 ± 4.79	5.90 ± 0.78	5.73 ± 0.55	102.45 ± 0.54	35.02 ± 0.31
TKA group	97.83 ± 8.26	85.62 ± 2.65	12.50 ± 1.41	6.95 ± 1.01	82.45 ± 0.54	30.56 ± 0.31
T value	−23.09	23.66	−25.90	−6.71	26.19	10.17
*P* value	**<0.01**	**<0.01**	**<0.01**	**<0.01**	**<0.01**	**<0.01**

Bold values indicate statistical significance.

cm, centimeter; d, days; g/L, grams per liter; min, minutes; ml, milliliters; TKA, total knee arthroplasty; UKA, unicompartmental knee arthroplasty.

### 3.4 Comparison of knee function scores

All patients successfully completed their surgeries, and postoperative imaging confirmed satisfactory prosthesis positioning and stability. The mean follow-up duration was 10.45 ± 1.98 months (range: 9–12 months) in the UKA group and 10.98 ± 1.98 months (range: 8–12 months) in the TKA group. In terms of complications, only one case of deep vein thrombosis (DVT) occurred in the UKA group (2.22%), while the TKA group reported seven complications: two cases of DVT, one superficial wound infection, three cases of fat liquefaction at the incision site, and one case of delayed wound healing, with a total complication rate of 17.5% (*P* < 0.05). There were no significant differences in VAS, HSS scores, or ROM between the two groups preoperatively (*P* > 0.05). At 1 and 6 months postoperatively, the UKA group showed significantly lower VAS scores (*P* < 0.01) and significantly higher HSS scores and ROM (*P* < 0.05), indicating superior early pain relief and functional recovery. However, by the 8th postoperative month, no significant differences were observed between the groups in these metrics (*P* > 0.05, Table 3).

**Table 3:** Follow-Up outcomes between the Two groups.

Indicator	Time point	UKA group	TKA group	T value	*P*-value
Visual analog scale (VAS, score)	Before Surgery	6.98 ± 0.92	6.80 ± 0.76	0.95	0.34
	1 months	3.42 ± 0.81	4.12 ± 0.21	−5.29	**<0** **.** **01**
	6 months	0.88 ± 0.72	1.48 ± 0.96	−3.16	**<0**.**01**
	8 months	0.31 ± 0.52	0.41 ± 0.12	−1.19	0.24
Hospital for special surgery score (HSS, score)	Before Surgery	47.08 ± 4.83	48.23 ± 4.64	−1.09	0.28
	1 months	70.15 ± 4.12	60.23 ± 4.23	10.63	**<0**.**01**
	6 months	80.88 ± 5.87	78.10 ± 4.93	2.29	**0**.**02**
	8 months	87.23 ± 3.12	86.23 ± 2.12	1.68	0.10
Range of motion (ROM, degrees)	Before Surgery	90.88 ± 4.97	91.03 ± 1.20	−0.13	0.90
	1 months	105.81 ± 4.25	98.03 ± 1.20	11.76	**<0**.**01**
	6 months	110.30 ± 4.13	103.40 ± 3.86	5.46	**<0**.**01**
	8 months	112.30 ± 4.13	111.35 ± 7.22	0.51	0.61

Bold values indicate statistical significance.

HSS, hospital for special surgery; ROM, range of motion; TKA, total knee arthroplasty; UKA, unicompartmental knee arthroplasty; VAS, visual analog scale.

## 4 Discussion

This study retrospectively analyzed data from patients with anteromedial osteoarthritis and Class II obesity (BMI 35.0–40.0 kg/m²), aiming to compare the early clinical outcomes of UKA and TKA and to provide a reference for surgical decision-making.

Obesity is an independent risk factor in knee arthroplasty: it increases postoperative complications and reduces long-term prosthesis survival. Moreover, although presurgical weight loss is recommended to optimize outcomes, many obese patients do not achieve meaningful or sustained weight reduction despite nutritional interventions and supervised rehabilitation programs (12, 13).

Compared to TKA, UKA requires minimal bone resection, disrupts less soft tissue, and preserves native knee structures, including both cruciate ligaments, thereby facilitating faster postoperative recovery, less pain, and improved early function (14). Nevertheless, UKA has traditionally been discouraged in obese patients due to concerns about endoprosthetic instability—loosening, subsidence, and insert dislocation—which raise revision risk. A study of 5,770 UKA cases reported significantly higher 90-day complication and revision rates in obese and morbidly obese patients vs. non-obese controls (15, 16).

With advancements in surgical precision, endoprosthetic design, and biomaterials (ultra-high-molecular-weight polyethylene and cast cobalt-chromium-molybdenum alloy), recent findings have supported the safety and stability of UKA in obese populations. Specifically, McElroy et al. reported that patients with morbid obesity (BMI ≥ 40 kg/m²) undergoing TKA had ^significantly^ lower endoprosthetic survival rates and Knee Society Scores compared to obese and non-obese groups, with complications occurring in 9%, 15%, and 22% of cases, respectively (*P* < 0.05) (17). These patients also experienced longer hospital stays, increased treatment costs, and were more likely to require post-discharge rehabilitation (18). Such findings underscore the perioperative risks and healthcare burden posed by TKA in patients with severe obesity.

### 4.1 Postoperative pain

Pain relief and enhanced quality of life are the primary goals of knee arthroplasty. Our study demonstrated significantly lower VAS scores in the UKA group at 1 and 6 months postoperatively (*P* < 0.01), indicating superior short-term pain control. Moreover, TKA often causes nighttime pain during the first postoperative month, leading to poor sleep quality, increased anxiety, impaired early rehabilitation, reduced ROM, and elevated thrombosis risk (19). UKA's less invasive nature and preservation of anatomical stabilizers (ACL, posterior cruciate ligament) may explain its better pain outcomes (10). For obese patients, postoperative pain may also be influenced by pre-existing chronic inflammation, soft tissue swelling, and mechanical overload. UKA's lower soft tissue disruption and more natural load distribution may be advantageous in these patients. Notably, by the 8th postoperative month, pain scores between groups were no longer significantly different (*P* > 0.05), suggesting that as tissue recovery progresses, pain perception in TKA patients improves. Surgical decisions should, therefore, consider patients’ pain tolerance, rehabilitation expectations, and functional demands.

### 4.2 Postoperative complications

The total complication rate was significantly lower in the UKA group (2.22%) than in the TKA group (17.5%) (*P* < 0.05). This may be attributed to factors such as lower surgical trauma and reduced blood loss. TKA requires extensive bone and soft tissue dissection, contributing to higher intraoperative blood loss: 97.83 ± 8.26 ml vs. 63.03 ± 4.76 ml in UKA (*P* < 0.01). Additionally, hidden blood loss in TKA can exceed 50% of total loss, with mean volumes reaching 1,400 ml (20). Although tranexamic acid is routinely used, it is less effective against hidden loss, increasing the risk of postoperative anemia (21), which can impair wound healing and raise infection risk. Our data also showed lower postoperative albumin levels in the TKA group (30.56 ± 0.31 g/L vs. 35.02 ± 0.31 g/L, *P* < 0.01), indicating greater metabolic stress, which may negatively affect immune function and tissue repair (22).

### 4.3 DVT

DVT occurred in 2.22% of UKA patients and 5.00% of TKA patients. Obese individuals often present with multiple chronic conditions, with studies showing that over 30% of obese TKA patients have ≥3 comorbidities vs. only 7% in non-obese counterparts (23). Longer operative times, greater soft tissue trauma, and prolonged immobility post-TKA contribute to venous stasis and thrombus formation (24, 25). Conversely, UKA allows for earlier mobilization, improving venous return and reducing thrombosis risk.

### 4.4 Wound-related complications

Currently, there is no consensus diagnostic standard; based on a literature review and our clinical experience, the following criteria were adopted: on postoperative days 5–7, the only symptom is increased wound drainage without other clinical signs; wound healing is poor with subcutaneous tissue separation and floating fat droplets visible in the exudate; there is no redness, swelling, or tenderness at the incision and no necrosis of the wound edges or subcutaneous tissue; and microscopic examination of the drainage reveals abundant fat droplets with three consecutive negative bacterial cultures.

The TKA group reported one, three, and one case of superficial infection, fat liquefaction, and poor joint capsule healing, respectively, while no such complications occurred in the UKA group. Larger incisions, multiple tissue layers, and greater wound tension in TKA contribute to poor healing, especially in obese patients with thick subcutaneous fat and compromised vascularity (15, 16, 22, 26) Notably, the need for broader exposure often leads to longer incisions, particularly for patellar eversion, further increasing the risk of healing-related complications (9, 27, 28). This study found that even with absorbable sutures, capsule healing issues occurred, suggesting that non-absorbable sutures may offer better wound stability, especially in high-risk obese populations.

### 4.5 Functional recovery and long-term considerations

UKA exhibited superior functional outcomes (HSS and ROM) at 1 and 6 months postoperatively (*P* < 0.05), but by 8 months the differences were no longer significant, suggesting TKA patients eventually achieved comparable results. While UKA yields better short-term recovery, concerns persist regarding long-term endoprosthetic stability; notably, insert dislocation—often due to imbalanced gaps, ligament laxity, or patient factors—remains a primary cause of UKA revision (29, 30). Importantly, obese patients are more prone to implant wear, subsidence, and failure due to higher joint loads. Some evidence suggests that TKA may offer superior durability and long-term outcomes in medial compartment KOA (31). However, large-scale analyses (Katanbaf RM et al.) (32) found no significant differences in 90-day, 1-year, or 2-year complication rates or TKA conversion risk at 2 and 5 years across BMI groups, supporting UKA as safe and feasible in patients with obesity. Mekkawy KL et al. (33) reported that although UKA patients with morbidly obesity have a slightly higher loosening risk, they experience fewer overall medical complications, lower readmission and infection rates, shorter hospital stays, and reduced costs compared to TKA, indicating UKA can be an option for carefully selected individuals with morbid obesity.

Furthermore, UKA success depends on surgeon experience: Kazarian et al. found that when UKA accounted for under 20% of knee arthroplasties, implant survival was merely acceptable, improving markedly at a 40%–60% volume (34–36). Even with strict indications, inexperienced surgeons had a UKA failure rate as high as 15% (37).

Therefore, surgical selection should be individualized based on the patient's joint condition, obesity level, activity expectations, and the surgeon's expertise. Future large-scale, multicenter, long-term studies are needed to validate the safety and effectiveness of UKA in obese patients and to refine perioperative management strategies.

### 4.6 Limitations

This study has several limitations. First, its retrospective design may introduce selection bias. Second, the sample size, although justified by *a priori* power analysis, remains modest and may limit subgroup analyses. Third, follow-up was limited to 1, 6, and 8 months—capturing only early functional recovery and complications. Previous literature shows that implant failures in obese patients often peak beyond two years; therefore, longer-term survivorship data (≥24 months) will be collected in future studies. Finally, all procedures were performed by a single surgeon, which may affect generalizability.

## 5 Conclusion

Both UKA and TKA effectively alleviate pain and improve knee function in AMOA patients with class II obesity. Although TKA affords superior long-term endoprosthetic durability, it entails longer recovery and higher perioperative complication rates. In contrast, UKA involves minimal surgical trauma, faster rehabilitation, and fewer incision-related complications, yielding clear early benefits. Therefore, for appropriately selected patients, UKA may offer advantages in early functional recovery and reduced perioperative morbidity compared with TKA.

## Data Availability

The original contributions presented in the study are included in the article/Supplementary Material, further inquiries can be directed to the corresponding authors.
