# Correction: Comparison of early outcomes between unicompartmental and total knee arthroplasty in patients with anteromedial compartment knee osteoarthritis and class II obesity: a retrospective case analysis

**DOI:** 10.3389/fsurg.2025.1728623

**Published:** 2025-11-03

**Authors:** Frontiers Production Office

**Affiliations:** Frontiers Media SA, Lausanne, Switzerland

**Keywords:** anteromedial osteoarthritis (AMOA), class II obesity, unicompartmental knee arthroplasty (UKA), total knee arthroplasty (TKA), early functional outcomes

The article type was erroneously given as Review. The correct article type is Original Research.

The Data Availability statement was erroneously omitted in the published article. The correct Data Availability statement is “The original contributions presented in the study are included in the article/Supplementary Material, further inquiries can be directed to the corresponding authors.”

The original version of this article has been updated.

